# Indigenous knowledge system associated with the uses of insects for therapeutic or medicinal purposes in two main provinces of Burkina Faso, West Africa

**DOI:** 10.1186/s13002-022-00547-3

**Published:** 2022-07-05

**Authors:** Mamadou Ouango, Rahim Romba, Samuel Fogné Drabo, Noufou Ouedraogo, Olivier Gnankiné

**Affiliations:** 1 Laboratoire d’Entomologie Fondamentale et Appliquée, Unité de Formation et de Recherche en Sciences de la vie et de la Terre (UFR-SVT), Université Joseph KI ZERBO, 03 BP, 7021, Ouagadougou, Burkina Faso; 2 Institut de Recherche en Sciences de la Santé, (IRSS), 03 BP, 7192, Ouagadougou, Burkina Faso

**Keywords:** Entomotherapy, Insect-derived products, Associated pathologies, Folk medicine, Traditional healing

## Abstract

**Background:**

Some insects are harmful to humans, plants and animals, but some of them can also be a source of proteins, fats, vitamins and minerals and be of therapeutic value. The therapeutic potential requires that medicinal insects and their derived products need to be scrutinized. This study highlights the indigenous knowledge related to their use of medicinal insects in peri-urban and urban areas of Burkina Faso.

**Methods:**

The survey was carried out among 60 traditional healers spread across two phytogeographical zones of Burkina Faso. The questionnaire focused on medicinal insects used by experienced traditional healers. Chi-square tests and principal component analysis were performed to test for significant differences regarding knowledge of how insects in phytogeographically different areas were used therapeutically in connection with different disease categories.

**Results:**

A total of 19 species of medicinal insects belonging to 6 orders were cited in connection with treatments of at least 78 pathologies and symptoms. Most frequently mentioned was gastroenteritis. Our study showed that 48.78% of the insects and their products were associated with 46 plant species for the treatment of pathologies. In addition, honey, beeswax and nests were the most widely insect products used.

**Conclusion:**

The current study allows us to identify medicinal insects as well as their products used in the treatment of pathologies and symptoms, suggesting the presence of a considerable diversity of therapeutically important insect species. These insects are used alone and/or with their products but often in association with medicinal plants. The results constitute a useful database for future studies of medicinal insects in central and western parts of Burkina Faso.

## Introduction

In terms of species, insects are the most numerous groups of living organisms. Up to now, more than one million species of insects have been described, comprising about 70% of all organisms [1]. Insects can be found in almost all habitat on earth and they interact with all components (abiotic and biotic) of their environments. Many of them are known to be destructive and harmful, and around 228 million cases of malaria accounting for 405,000 deaths (including 93% in Africa alone) are linked to *Anopheles* mosquitoes, major vector to malaria transmission [2]. *Sarcoptes scabiei*, not an insect, an arachnid belonging to arthropod, responsible of scabies diseases, affects about 300 million cases yearly worldwide [3]. Severe yield losses of crops amounting to 100,852. 85 ha per annum have been recorded due to caterpillars of the moth *Spodoptera frugiperda* infestations during the 2017/2018 agricultural campaign in Burkina Faso [4]. Some species of insects have even been involved in the destruction of a country’s infrastructure [5]. This is the case of *Coptotermes formosanus* that is an opportunistic feeder of any material containing cellulose. It is known to damage non-cellulose materials in search of food, including plastic, concrete and soft metal [6]. However, these various negative effects insects should not hide the insects’ undeniably useful roles in the ecosystem. Indeed, they balance the ecosystem for the following reasons: they play an important role in (i) Pollination (80% of the world's flowering plant species depend on entomogamy) [7], (ii) Scavenging, recycling and fertilizing, (iii) Positive interactions with the soil [8], (iv) Biological control [9], (v) Entomophagy as food for humans and animals [10], (vi) Providing economic benefits (marketing of products like honey, wax, silk, lac) and (vii) The treatment of disorders and diseases [11, 12]. For a long time, immemorial humans have used insects and their products for the treatment of various pathologies [12, 13]. Medicinal insects and their products can be used to treat many different diseases either directly or indirectly. Thus, honey bees and their products like honey, propolis, royal jelly, their venom, etc. can be used to treat different health problems [14, 15]. Insect therapy can be an excellent avenue for drug research regarding the great diversity in this group [11–13, 16–18]. This requires a deep knowledge of the medicinal insects, their chemical composition and their potential applications. However, if in countries like China, Korea, India or Brazil, many documents provide information on medicinal insects [14, 16, 18–22], this is not the case for many African countries and even more Burkina Faso. The existing data remained very scarce [8, 23] and need to be deepened. Our objective aimed at assessing local knowledge on medicinal insects associated with their potential utilizations in five localities from Burkina Faso.

## Methods

### Study areas

**Fig. 1:**
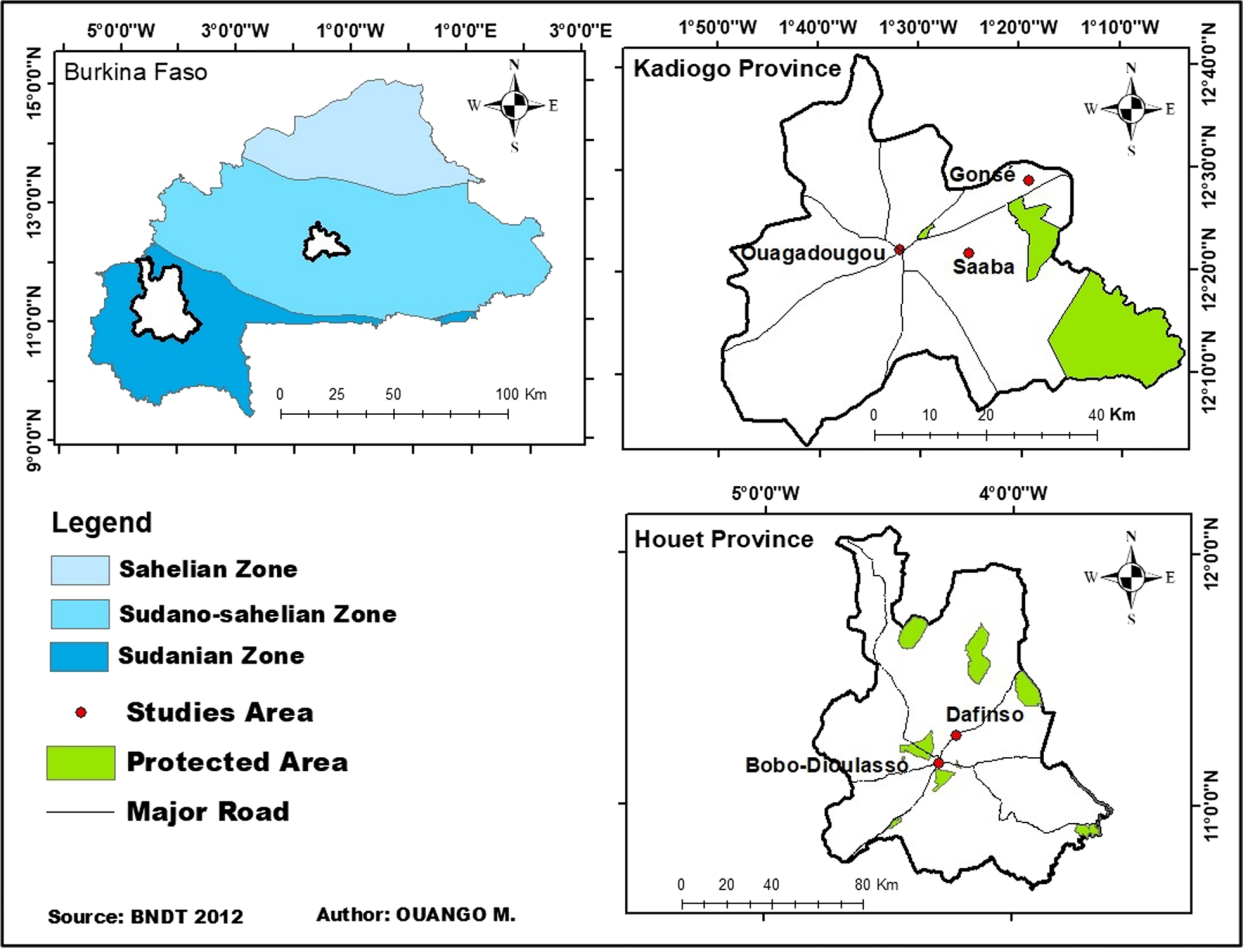
Map of the study areas indicating southern and northern Sudanese zones in Burkina Faso

This study was conducted from May to September 2020 in five localities across the Sudanian and Sudano-sahelian zones of Burkina Faso, located in the west part of the African continent. They are Bobo Dioulasso, Dafinso belonging to the province of Houët (9°–11°30′ N) and Ouagadougou, Saaba and Gonsé located in the province of Kadiogo (11°30′–14° N) (Fig. 1). The climate is tropical with two seasons: the dry (from October to April) and the rainy (from May to September) seasons in both study zones [24]. Mean annual rainfall ranged from 600 to 900 mm in the North Sudanian zone and 900 to 1000 mm in the South Sudanian zone (Fig. 1). The vegetation of the South Sudanian zone consists of a mosaic of savanna, dry forest and patches of gallery forests [25] and is characterized by Sudanian and Guinean species, whereas the North Sudanian zone is dominated by savanna with annual growing grass, trees, and shrubs [24, 25]**.**

### Data collection

Survey was carried out in traditional healers in the study area. In each province, 30 informants were interviewed through individual semi-structured interviews. Members of all ten ethnic groups were interviewed in both provinces. There were bissa, bobo, dafing, dioula, gourmantché, gourounsi, mossi, san, senoufo and turka without regarding their religious affiliation and their ages. Traditional healers were between 23 and 82 years old. A total of 60 traditional healers were interviewed in each site. The questionnaire included the photographs illustrating some medicinal insects and their products and also insects collection. During interviews or at a given period, insect specimens were collected and kept in bottles containing alcohol for identification according to Scholtz classification [26].

### Statistical analysis

Data processing and analysis were performed with the XLSTAT-Premium software 2016. Chi-square analysis was used to determine whether there were statistically significant differences among two climatic zones in knowledge of medicinal insects. Statistical significance was tested at the 5% level. Principal component analysis (PCA) was used to explore the variations in the medicinal insects use in different medical categories.

## Results

### Local knowledge extent on medicinal insects

#### Medicinal insects used and frequency of citations

**Fig. 2:**
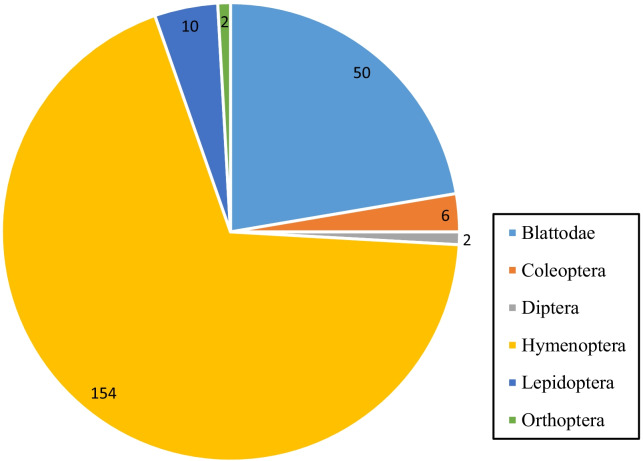
Citations by insects Orders in two climatic areas

**Fig. 3:**
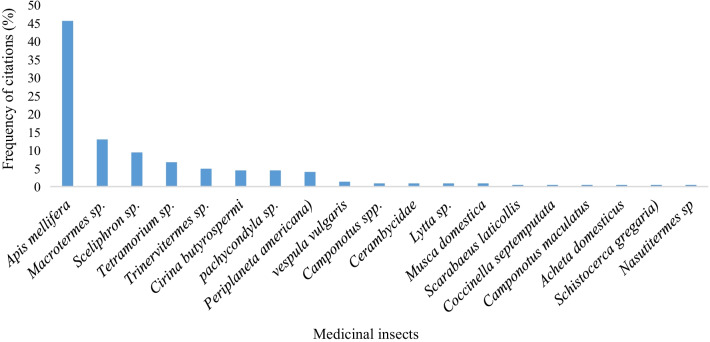
The frequency of citations of medicinal insects in two climatic zones

Nineteen (19) insect species belonging to six (6) orders that are Orthoptera, Blattodea known as hemimetabolous insects (exopterygota) and Hymenoptera, Coleoptera, Lepidoptera, and Diptera known as holometabolous insects (endopterygota) were cited as medicinal insects in the two climatic zones (Fig. 2). The cited medicinal insects belonging to Orthoptera were crickets (*Acheta domesticus*) and locusts (*Schistocerca gregaria*) with 0.45% frequency of citation per insect. As for the Blattodea, we had *Trinervitermes sp*., *Macrotermes sp.* tree-nesting termites (*Nasutitermes sp*.) and the cockroach (*Periplaneta americana*) with 4.91%, 12.95%, 0.45% and 4.02% as frequency of citation, respectively. Hymenoptera was honey bees (*Apis mellifera*), mason wasp (*Sceliphron sp*.), common wasp (*Vespula vulgaris*), carpenter ants (*Camponotus* spp.) hypoge nest ants (*Pachycondyla sp.)*, ground-nest ants (*Tetramorium sp*) and sugar ants (*Camponotus maculatus*) with 45.54%, 9.38%, 1.34%, 0.89%, 4.46%, 6.70% and 0.44% as frequency of citation, respectively. In Coleoptera, a longhorned beetles (Cerambycidae), acantharids (*Lytta sp.*), dung beetles (*Scarabaeus laticollis*), 7-spotted ladybirds (*Coccinella septempunctata*) belonging to Coleoptera were cited with 0.89%, 0.89%, 0.45%, at 0.45% of frequency of citation, respectively. Lepidoptera is represented by the shea caterpillar butterfly (*Cirina butyrospermi*) with 4.46% of frequency of citation. The Diptera was represented by flies (*Musca domestica*) with 0.89% of frequency of citation. (Fig. 3).

#### Comparison of medicinal insects used in the two climatic zones

**Fig. 4:**
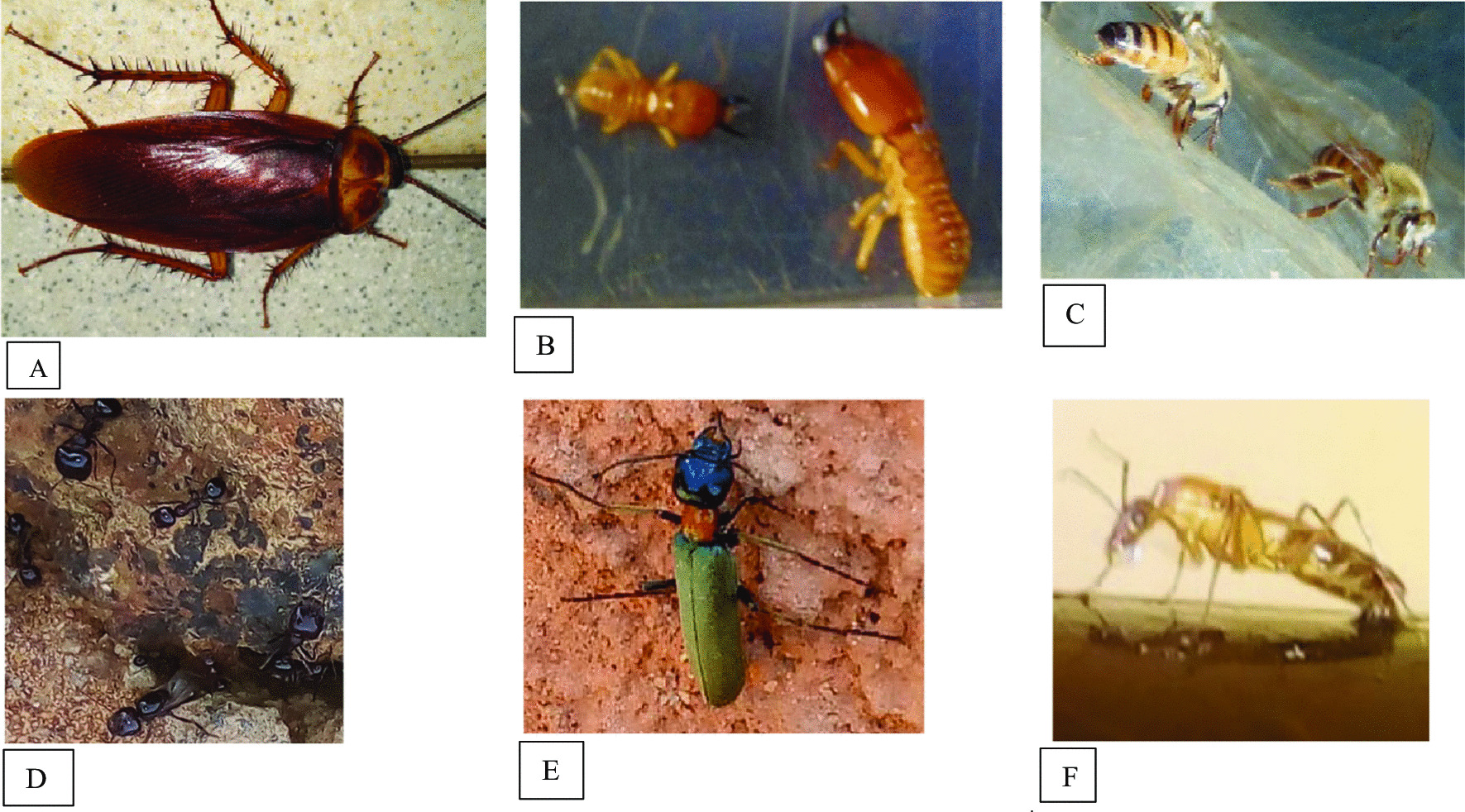
Some medicinal insects found in the two provinces: Kadiogo and Houët. **A** *Periplaneta americana*; **B** *Macrotermes sp.;* **C** *Apis mellifera*; **D** *Pachycondyla sp*.; **E** *Lytta sp;* **F** *Camponotus maculatus*

In both provinces, medicinal insects were used. However, the insects used to treat sick people are not always the same regardless climatic areas. *Periplaneta americana*) (A); *Macrotermes sp*. (B); *Trinervitermes sp.*; *Apis mellifera* (C), *Vespula vulgaris*; *Sceliphron sp*.; *Pachycondyla sp*. (D), *Tetramorium sp*.; *Lytta sp.* (E) and *Cirina butyrospermi;* were used in two climatic areas (Fig. 4). These medicinal insect’s species represented 52.63% of the cited species. However, five medicinal insects’ species were specific to the localities of the province of Houët. These are the cricket (*Acheta domesticus*), the carpenter ant (*Camponotus spp*.), the sugar ant (*Camponotus maculatus*) (F) (Fig. 4), the 7-spotted ladybird (*Coccinella septempunctata*), and the house fly (*Musca domestica*). As for the localities of the province of Kadiogo, the locust (*Schistocerca gregaria*), longhorn beetle (*Cerambycidae*), tree nest termites (*Nasutitermes*) and dung beetle (*scarabaeus laticollis*) were used specifically. However, the tests did not reveal a significant difference (Khi^2^ = 23.930, *P* = 0.2767) regarding the knowledge of medicinal insects in the two study areas. The difference in knowledge of traditional healers on medicinal insects is also not significant between sites in the Sudano-sahelian areas on the one hand and those in the Sudanian area on the other hand with respective Khi^2^ and p values of 13.407, 0.495 and 2.436, 0.494.

### Stages of development and insect-derived products used

#### Different stages of development of medicinal insects used

Medicinal insects were used at different stages of their development. Among the insects cited in the climatic areas, 13 of them representing 68.42% of the above-mentioned species were used at different stages of development to produce drugs. Thus, *Camponotus maculatus* was used at both pupae and imago stage, *Cirina butyrospermi* and *Musca domestica* at the larvae stage and the other ones at the adult stage. However, we have not recorded any medicinal insects used in the egg stage (Table 1).

**Table 1:** Developmental stages of insects used in therapy

Number of citations					
Insects/groups	Order	Egg	Larvae	Pupae	Adult
*Acheta domesticus*	Orthoptera	0	0	–	1
*Schistocerca gregaria*					
		0	0	–	1
*Periplaneta americana*	Blattodea	0	0	0	6
*Trinervitermes sp.*					
*Macrotermes sp.*					
		0	0	0	5
		0	0	0	4
*Apis mellifera*	Hymenoptera	0	0	0	2
*Camponotus spp.*					
*Camponotus maculatus*					
*Sceliphron sp.*					
		0	0	0	2
		0	0	1	1
		0	0	0	1
*Coccinella septempunctata*	Coleoptera	0	0	0	1
*Lytta sp.*		0	0	0	1
*Cirina butyrospermi*	Lepidoptera	0	10	0	0
*Musca domestica*	Diptera	0	1	0	1

#### Products derived from insects used in therapy

**Fig. 5:**
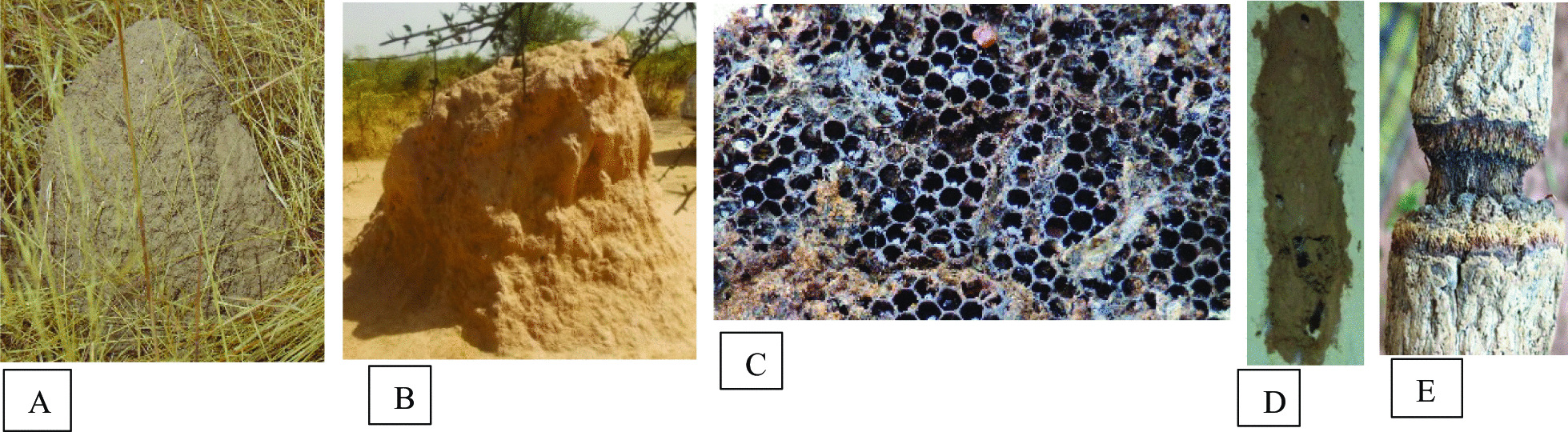
Medicinal product from insects. **A** *Trinervitermes sp*. nest, **B*** Macrotermes sp.* nest, **C** Beeswa*x*, **D** *Sceliphron sp*. nest, **E** Wood around the edge eaten away by a Cerambycidae

A rate of 52.26%, i.e., 10 of the insects cited were qualified as medicinal, because their products had the therapeutic virtues (Table 2). Nests and honey were the products most involved in the treatment of pathologies by traditional healers in the climatic areas with a frequency of citations of 44.16 and 42.42%, respectively. The honey and the wax (Fig. 5C) used were those of the honey bee. The nests used by traditional healers were from various insects: wasps (Fig. 5D), termites (Fig. 5A and B) and ants. There were also materials transformed by insects such as wood gnawed by longhorned beetles (Fig. 5E), the dung ball rolled by the dung beetle and the food accumulated by the ants in their nests.

**Table 2:** Medicinal products from insects

Number of citations					
*Insects/groups*	Order	Honey	Beeswax	Nests	Materials impacted
*Trinervitermes sp.* *Macrotermes sp.* *Nasutitermes sp.*	Blattodea	0	0	7	0
		0	0	33	0
		0	0	1	0
*Apis mellifera* *Pachycondyla sp.* *Tetramorium sp.* *Sceliphron sp.* *Vespula vulgaris*	Hymenoptera	98	27	0	0
		0	0	14	1
		0	0	18	0
		0	0	22	0
		0	0	4	0
*Scarabaeus laticollis* *Cerambycidae*	Coleoptera	0	0	0	1
		0	0	0	2

#### Medicinal insects and pathologies treated

Medicinal insects cited were involved in the treatment of 78 pathologies and symptoms. The various pathologies were grouped into 21 medical categories. Pathologies of gastroenterology and pulmonology were the most treated with medicinal insects in the two areas with the frequency of citations of 20.98% and 13.39%, respectively. While diseases of psychiatry, endocrinology and allergology with a frequency of citation of 0.4% per category were the less treated with insects and their products in these areas. One or more insects or products or the insect and its product may be used by a treatment of the given pathologies (Table 3). In 68.75% of cases, the pathology or symptom cited is treated by a single insect against 31.25% of cases where several insects were associated in the treatment of a given pathology. The diseases and symptoms treated by the same insects (Table 3) in these different localities were constipation, inflammation, difficulty in breathing, general fatigue, headache, cold, cough and vomiting.

**Table 3:** Insects and products use patterns in two provinces

Insect species (family)	Vernacular name	Insect or product	Preparation	Application	Disease cured	Used in combination with	Province
*Orthoptera*							
*1. Acheta domesticus*	sokɛɛrɛɛ (dioula)	Adult insect	NA	Oral	Deafness	NA	Houët
(Gryllidea)	buglunvãre (mooré)						
*2. Schistocerca gregaria*	sũurẽ (mooré)	Adult insect	Burnt insect powder	Topical	Wound		Kadiogo
(Acrididae)							
*Blattodae*							
*3. Macrotermes sp.* (Termitidae)	Yao-bi (mooré)	Nest	Powder	Topical	Knee pain	Decoction of *Cissus quadrangularis* branch	Houët
						Decoction of *Securidaca longipedunculata* branch	Kadiogo
				Oral	Diarrhea	Water	Houët
				Topical	Articular pain	Water	Houët Kadiogo
				Topical	Bone pain	*Datura stramonium*	Houët
				Topical	Sprain	Lemon juice	Houët
				Oral	General fatigue	–	Houët Kadiogo
				Topical	Fracture	–	Houët
				Oral	Gonorrhea	NA	Kadiogo
*3. Macrotermes sp.* (Termitidae)	Yao-bi (mooré)	Nest	Powder	Oral	Sexual impotence	NA	Houët
				Topical	Inflammation	Water	Houët Kadiogo
				Topical	Dislocation	Lemon juice + ash	Kadiogo
				Topical	Congenital	NA	Kadiogo
					malformation		
				Topical	Headache	NA	Houët Kadiogo
				Topical	General infertility	NA	Kadiogo
				Oral	Vomiting	–	Houët
						*Securidaca longipedunculata* branch decoction	Kadiogo
							Kadiogo
*4. Trinervitermes sp.* (Termitidae)	Tãmbeko (mooré)	Nest	Powder	Topical	Mumps	Crushed leaves of	Kadiogo
						*Guiera senegalensis*	
				Topical	Burn	*Acacia nilotica* bark	Houët
						decoction + Honey	
				NA	Iron deficiency	NA	Houët
				Topical	Fracture	Water	Houët
*4. Trinervitermes sp.* (Termitidae)	Tãmbeko (mooré)	Nest	Powder	Topical	Dropsy	Water	Kadiogo
				Topical	Inflammation	Water	Houët Kadiogo
				Topical	Edemas	Water	Houët
				Topical	Wound	NA	Kadiogo
				Oral	Vomiting	Water	Kadiogo
*5. Nasutitermes sp.* (Termitidae)	Ti-mogdo (mooré)	Nests	Powder	Topical	Inflammation	Water	Kadiogo
*6. Periplaneta Americana* (Blattidae)	Yalle (mooré) ɲɛbɛrɛ (dioula)	Adult insect	NA	NA	Headache	NA	Kadiogo
			Crushed live	Topical	Earache	–	Kadiogo
						Ash	Houët
			Burnt insect	Anal	Rectal prolapse	Shea Butter	Houët
			powder				
			Crushed live	Oral	Toxin	Unidentified plant bark	Kadiogo
			Crushed live	Topical	Shingles	*Guiera senegalensis* leaves + *Piliostigma*	Houët
						*reticulatum* roots decoction	
*Hymenoptera*							
*7. Apis mellifera*	Liden (dioula)	Insect adult	Powder	Oral	Sickle cell anemia	*Trichilia emetica* roots decoction	Houët
		Honey	NA		Cough	Tamarind fruit juice or *Diospyros mespiliformis* bark + *Calotropis procera* bark *or Tapinanthus sp.* branch decoction	Kadiogo
			NA		Cold	*Acacia albida* bark and leaves decoction decoction	Kadiogo
						Lemon fruit juice	Houët
			NA		General fatigue	*Bombax costatum* bark decoction	Kadiogo
						*NA*	Houët
			NA		Intestinal	NA	Kadiogo
					helminthiasis		
			NA		*Strangulated hernia*	*Cassia sieberiana* roots decoction	Houët
			NA		Sexual impotence	*Vitellaria paradoxa* flowers powder	Houët
			NA		Insomnia	–	Kadiogo
		Honey + beeswax	–		Memory loss	*Citrus Aurantiifolia* (fruit zest) *or Vitellaria Paradoxa* (flowers)	Houët
*7. Apis mellifera* (Apidae)	Liden (dioula)	Honey + beeswax	NA	Oral	Memory loss	*Piliostigma Thonningii*	Houët
						(leaves)*, or Cassia*	
						*Sieberiana (roots)* decoction	
		Honey	NA		Heart diseases	NA	Houët
			NA		Asthma	NA	Houët
			NA		Difficulty breathing	*Acacia albida* roots	Kadiogo
						decoction or *Curcuma longa*	
						crushed bulb	
						NA	Houët
			NA		Voice extinction	*Combretum micranthum* leaves decoction	Kadiogo
			NA		Cough	*Acacia nilotica* bark decoction	Houët Kadiogo
			NA	Oral	Pneumonia	Decoction of *Acacia albida* bark or *Boswellia dalzielii*	Kadiogo
						bark + *Acacia albida* bark + *Acacia nilotica* bark + *Glossonema boveanum* leaves + *Sterculia setigera* bark + *Brachystelma binger* (roots) decoction	
			NA	Oral	*Bladder lithiasis*	*Vitex agnus-castus* fruit	Kadiogo
			3 years old honey	Oral	Diabetes	NA	Houët
*7. Apis mellifera* (Apidae)	Liden (dioula)	Honey	NA	Oral	Constipation	*Terminalia avicenniodes* roots decoction	Houët
			NA	Topical	Burn	NA	Kadiogo Kadiogo
						*Acacia nilotica* bark	
			NA NA	NA Oral	Hemorrhage in women Nausea	Decoction of *Lannea acida* bark decoctio*n*	Houët Kadiogo
			NA	Oral	Pyrosis	*Cassia siberiana*	Kadiogo
			NA	Oral	Dizziness	NA	Houët
			NA	Oral	Toxin	*Zingiber officinale* crushed bulb + *Diospyros mespiliformis* bark decoction	Kadiogo
			–	Oral	Stomach aches	*Allium sativum or Annona senegalensis* + *Annona squamosa or Citrus aurantiifolia or Khaya senegalensis or Striga sp.*	Houët
*7. Apis mellifera* (Apidae)	Liden (dioula)	Honey Beeswax	–	topical	Foot pain	*Mangifera indica* leaves decoction	Houët
			Crushed wax	Oral	Gonorrhea		Houët
						*Lagenaria sicecaria* leaves decoction	
			Crushed	Oral	Ulcer	*Ocimum basilicum* leaves or	Kadiogo
			wax			*Acacia nilotica* fruit or *striga*	
						*sp. (*whole) or	
						*Cochlospermum tinctorium*	
						roots decoction	
						*Acacia nilotica* bark and leaves decoction	Houët Kadiogo
			Crushed	Topical	Itching	*Khaya senegalensis* bark decoction	Kadiogo
			wax				
			Crushed	Anal	Anal bleeding	NA	Houët
			wax				
			Crushed	Oral	Amenorrhea	*Ficus sycomorus*	Houët
			wax			*Ficus gnaphalocarpa* leaves	
						decoction	
7. *Apis mellifera* (Apidae)	Liden (dioula)	Beeswax	NA	Oral	General infertility	NA	Kadiogo
*8. Camponotus maculatus*	goɛtrgoɛɛga (mooré)	Adult insect and pupae	NA	Oral	Azoospermia	NA	Houët
(Formicidae)	folonfolonba (dioula)						
*9. Camponotus sp.* (Formicidae)	Sãati (mooré)	Adult insect	Powder	Topical	Foot pain	*Guiera senegalensis*	Houët
		Nest	Powder	Oral	Retention of acute urinary	NA	Houët
*10. Pachycondyla sp****.* (**Formicidae)	Gũuri (mooré)	Nest	Powder	Topical	Knee pain	Water	Kadiogo
				Oral	Headache	*Pupalia lappacea* crushed flowers	Kadiogo
						NA	Houët
				Oral	Stomach aches	*Cassia sieberiana* (roots) or *Guiera senegalensis*	Houët
						+ *Ficus polita* (roots) decoction	
				Topical	Neurological	*Khaya senegalensis* bark decoction	Houët
					problems		
				Oral	Retention of acute urinary	*Annona senegalensis* roots	Houët
					Retention of acute urinary		
						*Balanites aegyptiaca roots*	Kadiogo
				NA	Toxin	NA	Kadiogo
*10. Pachycondyla sp****.***	Gûuri (mooré)	Nest	Powder	Oral	Varicella	*Combretum molle*	Houët
*11. Tetramorium sp.* (Formicidae)	Kaya (mooré)	Nest	Powder		Sprain	NA	Kadiogo
				Topical	Inflammation	*Guiera Senegalensis*	Houët
						leaves decoction	
						–	Houët Kadiogo
				Oral	Cyst	*Acacia nilotica* roots	Houët
						decoction	
				Topical	Hip pain	NA	Kadiogo
				Topical	Headache	NA	Houët Kadiogo
				Topical	Neurological	*Khaya Senegalensis* dead bark decoction	Houët
					problems		
				Oral	Retention of acute urinary	*Annona Senegalensis* roots	Houët
						decoction	
				Oral	Gynecological problems	NA	Kadiogo
				Oral	Chronic cough	NA	Kadiogo
*12. Sceliphron sp.* **(**Sphecidae)	Vûnunvûnga (mooré)	Nest	Powder	Topical	Inflammation	*Xanthoxylum Zanthoxyloides* leaves decoction	Houët
						*–*	Houët Kadiogo
		Nest	Powder	Oral	Vomiting	*Tamarindus indica*	Kadiogo
						fruit juice	
				Topical	Allergy due to stings	*Xanthoxylum*	Houët
						*zanthoxyloides* leaves	
						decoction	
				Topical	Sprain	Water	Kadiogo
				Oral	Hiccups	NA	kadiogo
				Oral	Female	NA	Houët
					infertility		
				Topical	Lipoma	NA	Houët
				Oral	Sore throat	NA	Houët
				Topical	Hip pain	Water	Kadiogo
				Topical	Foot pain	NA	Houët
				Topical	Mumps	NA	Houët
				Topical	Fontanel	Water	Kadiogo
					problem		
				Oral	Cough	*Cassia sieberiana*	Houët
						roots decoction	
*13. Vespula vulgaris* (Vespidae)	Kãnenkãaga (mooré)	Nest	Powder	Topical	Lipoma	Lion fat	Kadiogo
				Oral	Heart diseases	NA	Houët
				Topical	Whitlow	NA	Houët
Coleoptera							
*14. Scarabaeus laticollis*	Gutungulungu	Rolled dung	NA	Oral	Painful urination	NA	Kadiogo
(Scarabaeidae)	(mooré)						
*15. Lytta sp.*	Pusg-n-waag-ma (mooré)	Adult insect	NA	Oral	Sickle cell anemia	*Ficus sycomorus*	Houët
						*Ficus Gnaphalocarpa* bark	
						and roots decoction	
			NA	Oral		NA	Kadiogo
					Retention of acute urinary		
*16. Coccinella septempunctata*	–	Adult insect	Burnt insect powder	Topical	Wound	NA	Houët
(coccinellidae)							
17. Unidentified specie (Cerambicidae)	Ti-gẽnengẽega (mooré)	Insect gnawed wood	Ash of wood	Topical	Breast crack	NA	Kadiogo
*Lepidoptera*							
*18. Cirina butyrospermi (*Saturniidae)	Pilimpiuku (mooré)	Larvae	Cooked larvae	Oral	Asthma	*Parkia biglobosa* cooked seed	Kadiogo
					Arterial	NA	Houët Kadiogo
					hypertension		
					Avitaminosis	NA	Houët
					Abdominal bloating	NA	Kadiogo
					Diabetes	NA	Houët
					Rage	NA	Houët
					Tetanus	NA	Houët
					Toxin	NA	Kadiogo
Diptera							
*19. Musca domestica* (Muscidae)	Limɔgɔ (dioula)	Adult insect	NA	Oral	Sickle cell	*Cassia sieberiana* bark and	Houët
					anemia	Roots decoction	
		Larvae	NA	Oral	Male infertility	NA	Houët

*NA*: not applicable

#### Distribution of medicinal insects by medical category

**Fig. 6:**
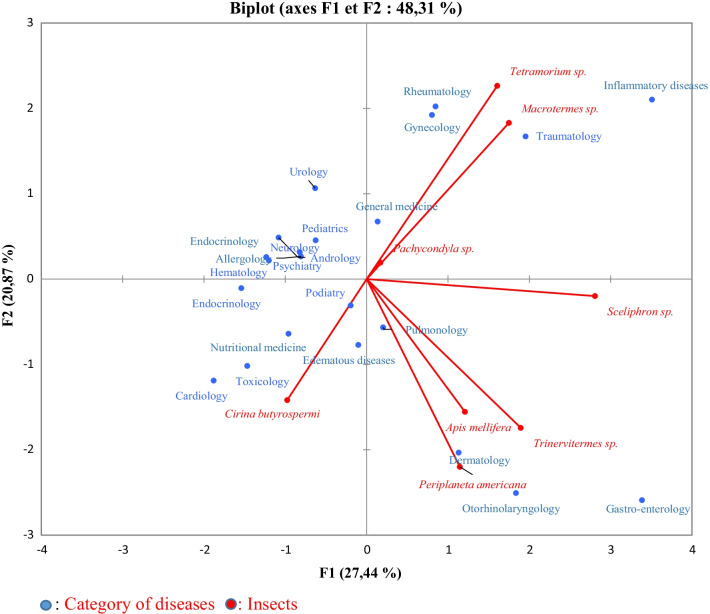
Distribution of medicinal insects according to the medical category in the PCA plan

The insects used in the different medical categories were subjected to a principal component analysis (PCA) (Fig. 6). The analysis revealed that the first two components explained 48.31% of the variability observed within the surveyed population. PCA showed medical categories treated by *Cirina butyrospermi* (Lepidoptera), opposite to *Macrotermes sp*., Trinervitermes sp., *Periplaneta Americana* (Blattodea), *Apis mellifera Sceliphron sp*, *Pachycondyla sp.* and *Tetramorium sp.* (Hymenoptera).

#### Different associations between medicinal plants and insects in therapy

In 46.88% of cases, insects were associated with medicinal plants in the treatment of pathologies. Forty-six plant species associated with insects were reported in various treatments advised by traditional healers in the two provinces. The part of plants concerned were both grasses and woody plants. However, insects were not associated only with plants but also with either mineral substance including ash with 0.45% as frequency of citation or fat of animal such as lion's fat (0.45% frequency of citation) for certain treatments. Survey showed that some medicinal insects are involved in the same disease treatments in both localities. Overall, the kind of association of medicinal insects with plants varied from one province to another. Globally, *Apis mellifera* was most medicinal insect associated with various plants that targeting a large spectrum of pathologies (Table 3).

## Discussion

### Local knowledge regarding medicinal insects

#### Medicinal insects used

Our study revealed nineteen (19) medicinal insects used by traditional healers, showing very rich ethnomedicine knowledge in the two provinces of Burkina Faso. There are similarities with other studies carried out in the world generally, and in Africa specifically, where bees (Hymenoptera) and their products, but also beetles (Coleoptera) and cockroaches (Blattodea), were predominant in the list of the therapeutic species [11, 14, 18]**.** Thus, insect and insect-derived products provide ingredients that have been a staple in traditional medicine for centuries in many parts of the world. In fact, their immunological, antiviral, analgesic, antibacterial, anti-cancer, diuretic, anesthetic, antioxidant, anti-inflammatory, anti-rheumatic and immunomodulatory properties are well recognized [12, 27, 28]. The use of medicinal insects varied from one locality to another and also from one country to another. Then, the adult house cricket, *Acheta domesticus* (Gryllidae), is used for the treatment of deafness in Burkina Faso while in Latin American, it is used for the treatment of scabies, asthma, eczema, lithiasis, earache, oliguresis, rheumatism, urine retention, urinary incontinence and ophthalmological problems [29]. As for locus *Schistocerca gregaria* (Acrididae), it is used by the traditional healers in Burkina Faso to treat wound. This insect is known to have antiproliferative activity [30–32] The Blattodae, *Periplaneta americana,* is used by the traditional healers in Burkina Faso to treat ear pain, but the same species has been used to treat asthma, toothaches and bronchitis by the Amerindians of the Amazon [33]**.** It is also used for an asthma treatment in Latin American folk medicine [29]. This property to treat pain is probably due to the presence of molecules isolated from the brains of these insects known to be excellent antibiotics v[34]**.** The therapeutic practice to use blister beetle *Lytta vesicatoria* for the treatment of urinary retention has also been reported in other studies undertaken by Read et al. [35]*.* and Tsuneo et *al.* [36]. These common uses are probably certainly due to the presence of cantharidin, a compound with notable effects on the urogenital system of vertebrates. In the past, it was prescribed as a remarkable aphrodisiac, but now it is used to induce mating in some domestic animals and as a therapy for some disorders of the urinary tract [14]. Furthermore, a longhorned beetle (Cerambycidae), dung beetle (*Scarabaeus laticollis)*, and 7-spotted ladybirds (*Coccinella septempunctata)* are used to treat, respectively, breast crack, pain on urination and wound. The larvae of Diptera *Musca domestica* are used to treat sickle cell anemia and male infertility, respectively, in the province of Houët. As for Lepidoptera, the larvae of *Cirina butyrospermi* are recognized to have tonic properties in the study areas, as also noted by Oudhia et *al.* [37]. Its larvae have an ability to regulate blood pressure in hypertension. The fact that insect species are being used for the same purpose by several communities might indicate their pharmacological effectiveness. The widespread use of insects throughout the world suggest that traditional knowledge on zootherapy is to be studied more seriously, in order to lead to the discovery of new sources of drugs [38].

#### Stage of development of medicinal insects’ use

Overall insects are used at different stages of development. However, in our study, the egg stage was not cited. Larvae, pupae and adults’ stage have nutritional and medicinal qualities [39]. These authors point out that in general, the protein content was found to be higher along with the more mature developmental stages. Honeybee larvae were used for the treatment of male impotence and for raising libido in men. These are usually consumed directly within the wax combs. The use of larvae for treating infertility is probably due to their high protein content of mature larvae (15.4% of fresh weight) [40]. For *Musca domestica*, larvae are used in treating male infertility whereas in Japan, it is used in treating snake bites and fever, gut and stomach problems and eye disorders [12].

#### Insect products used in therapy

The insect products cited in our study are the nests of termite and honey and wax of bee. However, other results suggest, in addition to the bee products mentioned above, propolis and royal jelly [41]. Honey is the most widely used as bee product in traditional medicine and its use varies by region. This could be explained by the variation in the composition of honey depending on the region. Indeed, the composition of honey and its content of mineral and organic constituents are strongly linked to foraged flowers [8]. In addition to these products known to medicinal insects, the study showed that insects can impact certain materials and give them therapeutic benefits. This transformation could come from the secretions of these insects. Indeed, insect secretions have been shown to have therapeutic properties as regarding secretions from larvae of *Lucia sericata* [42, 43]. The nests of termite *Macrotermes spp*. and *Trinervitermes spp.* are used to treat diarrhea, fractures and used for its toning effect and those of *Nasutitermes spp.* is used as an anti-inflammatory activity. Healing properties of termite mounds could be explained by the fact that they contain xyloglucan, a hemicellulose in the wall of dicotyledons that reduces the frequency and duration of diarrhea [44]. Undegraded sugars present in termite droppings could explain their use as plaster to immobilize fractured limbs [45]. Interestingly, *Apis mellifera, Vespula vulgaris, Sceliphron sp* belonging to Hymenoptera are listed among medicinal insects and their products are used to treat different diseases around the world. Thus, the nest of *Sceliphron sp*. is used to treat mumps. As to *Apis mellifera*, besides honey used to treat asthma, burn, constipation, difficulty breathing, voice extinction, general fatigue, insomnia, intestinal helminthiasis, bladder lithiasis, heart diseases, and hip pain, other bee products are highly prized as medicines. Pollen (collected by bees), larvae and pupae have medicinal properties, i.e., pollen is used for the treatment of bleeding gastric ulcer and chronic prostatitis [46–48]. Propolis, which is a resinous substance collected from the buds of some trees and flowers by bees to repair damage to their hives, is used in Eastern Europe as an antiseptic and an anti-inflammatory agent for the treatment of wounds and burns [49].

#### Correlations between medicinal insects and medicinal categories

Insects are used in the treatment of a wide variety of pathologies and symptoms. This broad spectrum of insect action could be understood if we consider the extreme variability of individuals of this class. Also, we can think of a great variability of the active molecules that can be contained in these different insects and products. PCA revealed a strong positive correlation between *Cirina butyrospermi* larvae and nutritional diseases. Indeed, shea caterpillars are very rich in protein (63%), but also in omega 3, iron, zinc, magnesium, phosphorus (5%) and vitamins A, D, E [50]. PCA has also shown that honey from bees is widely used in the treatment of gastroenterological pathologies. Indeed, it has been revealed that bee products can regulate digestive disorders (diarrhea, colitis, peptic ulcer) induced by the bacterium *Helicobacter pylori* [51]. Honey can be a complementary treatment for bacterial gastroenteritis in children [52]. This same PCA testified the use of *Macrotermes sp*. particularly its nest in the treatment of pathologies of rheumatology and gynecology. Other studies have also shown the implication of this insect's nest in the treatment of disorders related to human reproduction. Indeed, Zborowski [53]confirmed in his study that the queens of *Macrotermes sp.* were believed to have the power to treat female infertility and male impotence. This nest is also used against inflammatory diseases as it has been shown in Mahdi et *al.* [54].

#### Association between medicinal insects and plants

As for the association between insects and plants for the treatment of pathologies in the two study areas exhibits many variabilities. This fact could be explained by the different floristic knowledge of traditional healers in the different study areas. Here, plants (flowers, fruits, leaves, barks and roots) were added to insect and their products, either or adjuvant and therapeutic.

## Conclusion

Insects or their products have therapeutic virtues affecting several categories of modern classical medicine. The predominant order cited in this current study is Orthoptera, Blattodea (exopterygota) and Hymenoptera, Coleoptera, Lepidoptera, and Diptera (endopterygota). Also, insects and products are used alone or in combination with ash, fat or with various organs of flowering or non-flowering plants. The predominantly used insect products are termite nests and bee honey in the two study areas. Honey is mainly used in the therapy of gastroenteritis and termite nests in the treatment of inflammatory and trauma diseases. The treatment of pathology in which an insect is used depends on the product with which it is combined and on the region. In fact, insects are used differently in most cases in the different survey areas. This study provides a new insight of medicinal insects and open new avenues for their putative valorization in Burkina Faso.

## Data Availability

Not applicable.
